# Integrating biophysical modeling, quantum computing, and AI to discover plastic-binding peptides that combat microplastic pollution

**DOI:** 10.1093/pnasnexus/pgae572

**Published:** 2025-02-18

**Authors:** Jeet Dhoriyani, Michael T Bergman, Carol K Hall, Fengqi You

**Affiliations:** Systems Engineering, College of Engineering, Cornell University, Ithaca, NY 14853, USA; Department of Chemical and Biomolecular Engineering, North Carolina State University, Raleigh, NC 27606, USA; Department of Chemical and Biomolecular Engineering, North Carolina State University, Raleigh, NC 27606, USA; Systems Engineering, College of Engineering, Cornell University, Ithaca, NY 14853, USA; Robert Frederick Smith School of Chemical and Biomolecular Engineering, Cornell University, Ithaca, NY 14853, USA; Cornell University AI for Science Institute, Cornell University, Ithaca, NY 14853, USA

**Keywords:** microplastics, peptide discovery, biophysics, quantum computing, artificial intelligence

## Abstract

Methods are needed to mitigate microplastic (MP) pollution to minimize their harm to the environment and human health. Given the ability of polypeptides to adsorb strongly to materials of micro- or nanometer size, plastic-binding peptides (PBPs) could help create bio-based tools for detecting, filtering, or degrading MNP pollution. However, the development of such tools is prevented by the lack of PBPs. In this work, we discover and evaluate PBPs for several common plastics by combining biophysical modeling, molecular dynamics (MD), quantum computing, and reinforcement learning. We frame peptide affinity for a given plastic through a Potts model that is a function of the amino acid sequence and then search for the amino acid sequences with the greatest predicted affinity using quantum annealing. We also use proximal policy optimization to find PBPs with a broader range of physicochemical properties, such as isoelectric point or solubility. Evaluation of the discovered PBPs in MD simulations demonstrates that the peptides have high affinity for two of the plastics: polyethylene and polypropylene. We conclude by describing how our computational approach could be paired with experimental approaches to create a nexus for designing and optimizing peptide-based tools that aid the detection, capture, or biodegradation of MPs. We thus hope that this study will aid in the fight against MP pollution.

**Significance Statement**

Microplastics (MPs), defined as plastic particles smaller than 5 mm, are a concerning environmental pollutant. The ability of peptides to adsorb micro- and nanoscopic materials suggests that peptides could help remediate MP pollution. Here, we combine biophysical modeling, quantum computing, and reinforcement learning to discover peptides that bind strongly to common plastics. Simulations revealed that the discovered peptides have high affinity for polyethylene and polypropylene. The peptides found in this work can potentially be used in biological tools to remediate MP pollution.

## Introduction

It is imperative to develop methods for detecting and capturing microplastic (MP) pollution due to its environmental and health concerns (1–4) and the regular consumption of MPs by humans (5). While a variety of methods have been developed (6–10), a promising tool is peptides. Both proteins (11–16) and peptides (17–21) adsorb to many materials, including plastics. Peptide adsorption can be strong, with adsorption free energies in the range of 5 to 15 kcal/mol for various materials (20, 22–25). These findings suggest that plastic-binding peptides (PBPs) could be used to help detect and/or capture MPs. PBPs may be especially useful for remediating nanoplastics since peptides adsorb rapidly and strongly to nanomaterials (26–29). Despite the promise of PBPs for MP remediation, they have received little attention in the literature except for a few studies (30, 31). We believe this is primarily due to the lack of PBPs for most common plastics. If PBPs were available, then peptide-based MP remediation strategies could be developed more readily.

How can PBPs be discovered? Peptides with affinity for solids, including polystyrene (PS) (32–35) and polypropylene (PP), can be discovered through library screening, in which a vast number of peptides are evaluated via a high-throughput experimental method. Library screening has limitations, though. It samples only a small fraction of possible peptide sequences, does not quantify peptide affinity, and provides no insight into why some peptides bind more strongly than others. We posit that PBPs can be more effectively discovered by introducing biophysical modeling and computational optimization alongside experimental methods. Modeling can quantify PBP affinity and describe how PBP affinity depends on environmental conditions or MP properties; optimization tools can leverage the data and insights generated by modeling to discover PBPs tailored to a target set of conditions.

The biophysical model should accurately predict peptide affinity while being computationally cheap enough to permit large-scale sampling of peptides. A suitable balance is offered by MM/GBSA (36), which is both fast and models peptide–plastic interactions at atomic resolution. However, MM/GBSA has notable simplifications: it uses an implicit solvent model and does not fully account for the configurational entropy of flexible molecules like short peptides. It thus is essential to validate PBPs using molecular dynamics (MD) simulations, a well-established method for calculating peptide affinity to solids (23, 24, 37–39).

Of the many computational optimization tools that can be applied to peptide discovery, we chose quantum annealing (QA) and reinforcement learning. Solid-binding peptides have been computationally discovered by applying bioinformatics tools to peptide library screening data (40–42). However, the small size or lack of datasets on PBPs prevents the use of bioinformatics tools. A second approach is to combine biophysical modeling with classical optimization tools like simulated annealing (43, 44) or genetic algorithm (45, 46), which search for minima on the energy surface defined by the model (47, 48). For example, simulated annealing was used by PepBD to discover PBPs (49). However, we suspect that QA could be a better choice. QA is designed to find the optima of Potts models and may solve large combinatorial problems that are difficult for classical computational methods (50). These useful attributes motivated past applications of quantum computing to peptide discovery and modeling (51, 52). However, both classical and quantum optimization methods do not “learn” from their sampling. This is a concern since only a tiny fraction of possible peptide sequences are sampled in a reasonable computational time, which makes intelligent sampling crucial for peptide design. Intelligent sampling is offered by generative AI tools (53–56), which have shown success in discovering antimicrobial peptides (57–60), anticancer peptides (61–64), cell-penetrating peptides (50, 65), and self-assembling peptides (51, 52). We specifically choose proximal policy optimization (PPO), a reinforcement learning method, because it effectively navigates high-dimensional sample spaces, such as amino acid sequences (66–68).

In this work, MM/GBSA modeling, MD simulations, QA, and PPO were combined to identify and evaluate PBPs. We first formulated a Potts model that expresses peptide affinity for a given plastic as a function of the amino acid sequence for a fixed conformation of a peptide adsorbed to a plastic surface. PBPs were discovered by using QA to find the amino acid sequence with the best Potts model score for many different conformations. This produced PBPs for four types of plastics commonly found in MP pollution: polyethylene (PE), PP, PS, and polyethylene terephthalate (PET). Calculation of the PBP adsorption free energy in MD simulations showed that the discovered PBPs for PE and PP have comparable affinity to recently discovered PBPs (49). PBPs for PS and PET had poor affinity, which indicates that the biophysical Potts model requires tuning. The Potts model was also solved using PPO to find PBPs for PE with an even broader range of physicochemical properties. Interestingly, PPO sampling also shed light on the sequence-structure relationship by relating the amino acid type that optimizes affinity for PE to the location of the side chain with respect to the plastic surface and the rest of the peptide. We conclude by describing how our computational strategy can be integrated with experimental methods to create a nexus that can develop and optimize peptide-based strategies to capture, detect, or degrade MPs. Overall, this work is an important step in developing biological tools for remediating MP pollution.

## Results

### Creating a Potts model that expresses peptide affinity for plastic as a function of the amino acid sequence

**Fig. 1.**
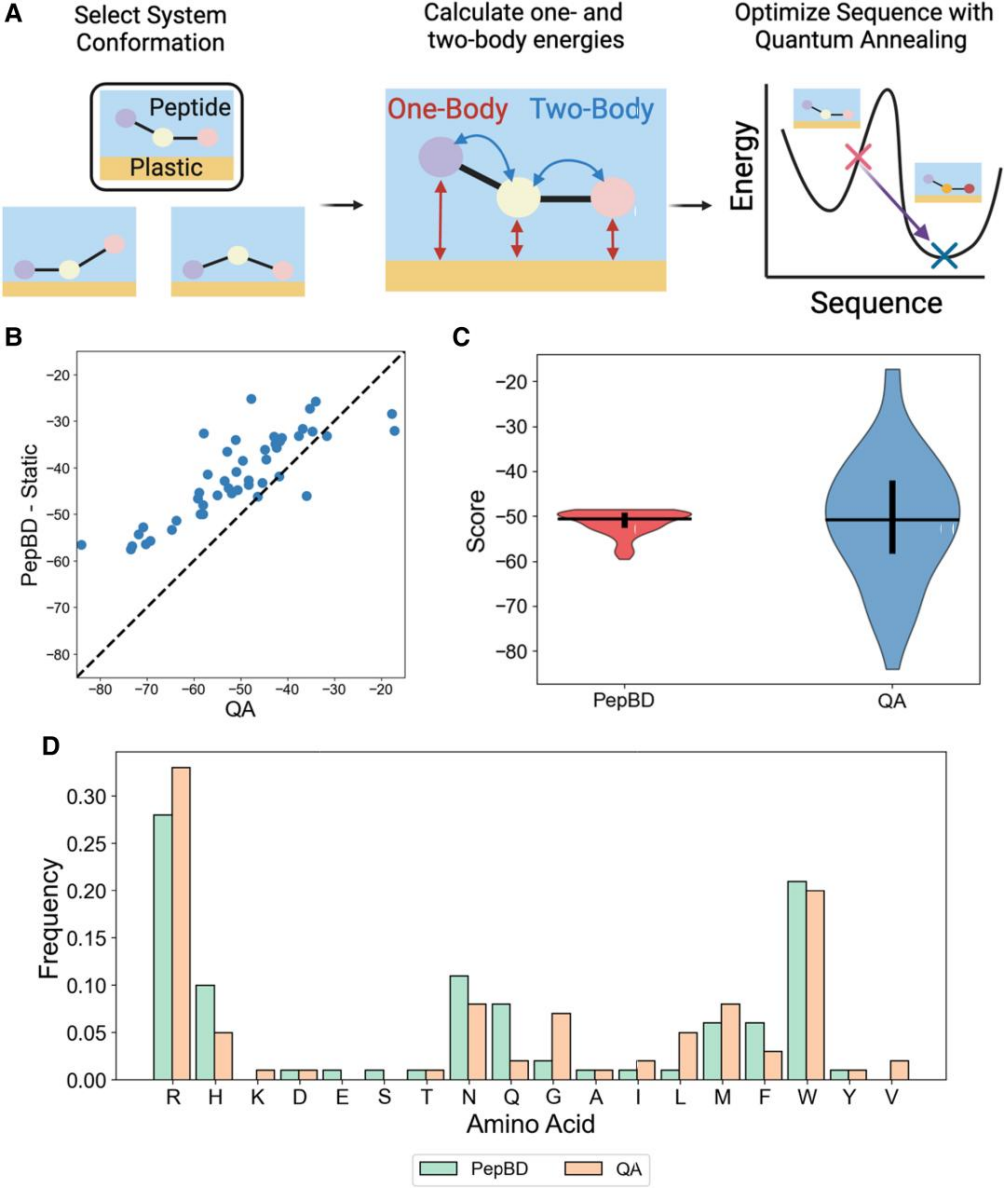
Outline of PBP discovery process and results for PE-binding PBPs. A) Schematic of pairing a Potts model and QA to discover PBP. Figure created using Biorender. B) Comparison of the best scores from QA and PepBD-Static for 50 system conformations. Each point corresponds to one system conformation, where the QA score is the *x* value and the PepBD-Static score is the *y* value. Points lie on the y=x dashed line if QA and PepBD-Static performed equally well; points lie above the line if QA found a better score than PepBD. C) Comparison of PBP scores from PepBD and QA. PepBD data were taken from previous work (49) and contain 100 sequences. D) Amino acid frequencies of QA and PepBD-Static PBPs for the 50 system conformations in B).

To discover PBPs with high affinity for plastic, we formulated a Potts model (69) that expresses peptide affinity for plastic as a function of the amino acid sequence (Fig. 1A). A PBP can then be found by finding the minimum of the Potts model. A sketch of the formulation is provided here, and details can be found in Materials and Methods. The Potts model takes the form


(1) $$\mathrm{Score}\;=\;\underset{i,\alpha }{\sum }\;s_{i\alpha }E_{i\alpha }+\lambda \underset{i,\alpha }{\sum }\;\underset{\;j>i,\beta }{\sum }\;s_{i\alpha }s_{\;j\beta }E_{i\alpha ,j\beta }$$

where $s_{i\alpha }$ is a binary variable that equals 1 if amino acid type *α* is at residue *i*, and 0 otherwise. Thus, *i* and *α* iterate over the peptide residues and the amino acid options, respectively. All peptides in this work have 12 residues and use all natural amino acids except proline. The one-body energy, $E_{i\alpha }$, is the interaction of amino acid type *α* at residue *i* with the plastic surface and itself. The two-body energy, $E_{i\alpha ,j\beta }$, is the interaction energy between amino acid type *α* at residue *i* and amino acid type *β* at residue *j*. Thus, the first term in (1) primarily optimizes peptide affinity for the plastic and the second term ensures that the peptide structure is stable (70). Finally, *λ* is a scaling factor that controls the relative importance of peptide affinity and peptide stability. By calculating all $E_{i\alpha }\;$ and $E_{i\alpha ,j\beta }$ values, the Score for any amino acid sequence can be calculated by summing all precalculated values. PBPs can then be discovered by finding the sequence with the lowest Score, where a lower Score corresponds to greater predicted affinity. It is important to note that $E_{i\alpha }$ and $E_{i\alpha ,j\beta }$ depend on the system conformation (i.e. the peptide backbone and the relative position and orientation of the peptide with respect to the plastic surface), which means that the lowest scoring sequence depends on the conformation. We thus use many initial conformations, with the initial conformations obtained from MD simulations. The best-scoring PBPs for all system conformations are combined to give the final set of putative PBPs for the target plastic.

### Pairing QA with the Potts model to discover PE-binding peptides

PBPs for PE were discovered by using QA to find the sequence with the lowest binding energy for a given Potts model. We first focus on PE to test our strategy, and search for PBPs for other plastics later. As a reference point for our designs, we also searched for PBPs using PepBD, a method recently used to find PBPs that also uses MM/GBSA to calculate peptide affinity for plastic (49). Since the system conformation must be fixed and the Potts model is only valid for a static peptide structure, PepBD conformation moves (71) were disabled in this comparison. We term this variant of PepBD as “PepBD-Static.” Comparison of the best scores between QA and PepBD-Static over fifty unique starting conformations shows that QA consistently found better-scoring sequences (Fig. 1B). The score difference between QA and PepBD-Static increases as the score of QA sequences decreases, a significant feature since the sequences with the lowest scores are the most promising PBPs. However, PepBD outperformed QA for six conformations, and QA yielded positive scores for three of these conformations. The poor performance may be due to suboptimal hyperparameters (see Materials and Methods for discussion) but is not critical for PBP discovery: the PepBD-Static scores for these six conformations are poor relative to the other system conformations, so these system conformations do not give rise to promising PBPs that should be further evaluated. As a second evaluation of our design strategy, the scores of the discovered PBPs were compared to the best PBPs previously obtained using PepBD where conformation changes were allowed (49) (Fig. 1C). While PepBD peptides have less variability in their score, the best QA peptides have better scores. Thus, our approach for discovering PBPs for PE appears successful. It is notable that QA and PepBD peptides have very similar amino acid compositions even though QA consistently performs better (Fig. 1D). The similar composition implies that QA outperforms PepBD-Static not by altering the amino acid composition, but by finding more optimal arrangements of the same amino acids.

**Fig. 2.**
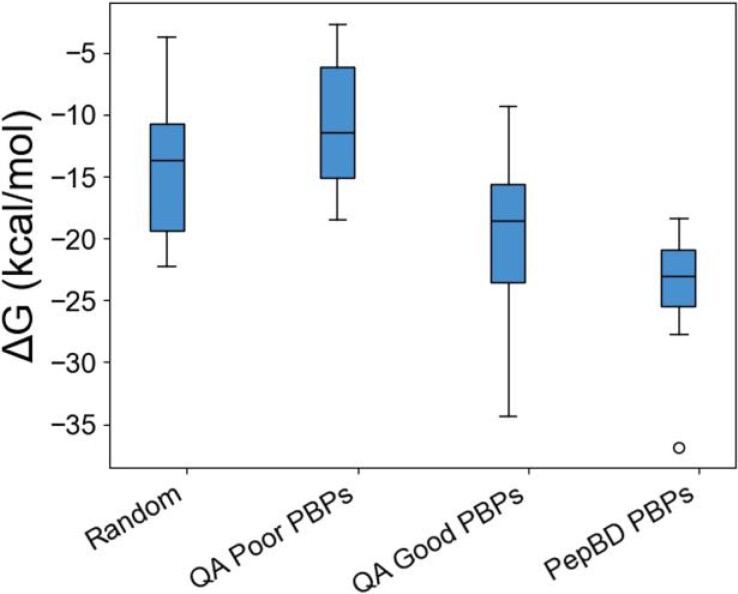
PBPs for PE found by QA shown to have high affinity in MD simulations. Binding free energy (ΔG) distributions for randomly generated sequences (12 peptides), “QA Poor PBPs” with a score >−50 (16 peptides), “QA Good PBPs” with a score <−50 (16 peptides), and the best PepBD PBPs obtained in previous work (49) (20 peptides).

MD simulations show that the best PBPs found by QA for PE have equal affinity as PBPs previously found by PepBD (49). Since the peptides found by QA have scores that span a large range (Fig. 1B), the PBPs were split into two groups: “Good QA PBPs” with scores <−50 and “Poor QA PBPs” with scores >−50. The affinity of the PBPs was evaluated by comparing to the 20 best PBPs previously obtained with PepBD (49) and 20 peptides with randomly generated amino acid sequences. PepBD peptides were chosen rather than PepBD-Static peptides since the former have greater predicted affinity and thus are a more stringent comparison. Good QA PBPs had equal or slightly greater affinity for PE than the best PepBD peptides and much greater affinity than random peptides (Fig. 2). In contrast, poor QA PBPs have roughly equal affinity to the random peptides and lower affinity than the PepBD PBPs. These results illustrate two key points: (ⅰ) PBPs with high affinity for PE were discovered by combining the Potts model and QA and (ⅱ) many system conformations are needed to effectively find PBPs because not all system conformations lead to high-affinity peptides.

### Discovering PBPs for multiple plastics by combining QA with the Potts model

**Fig. 3.**
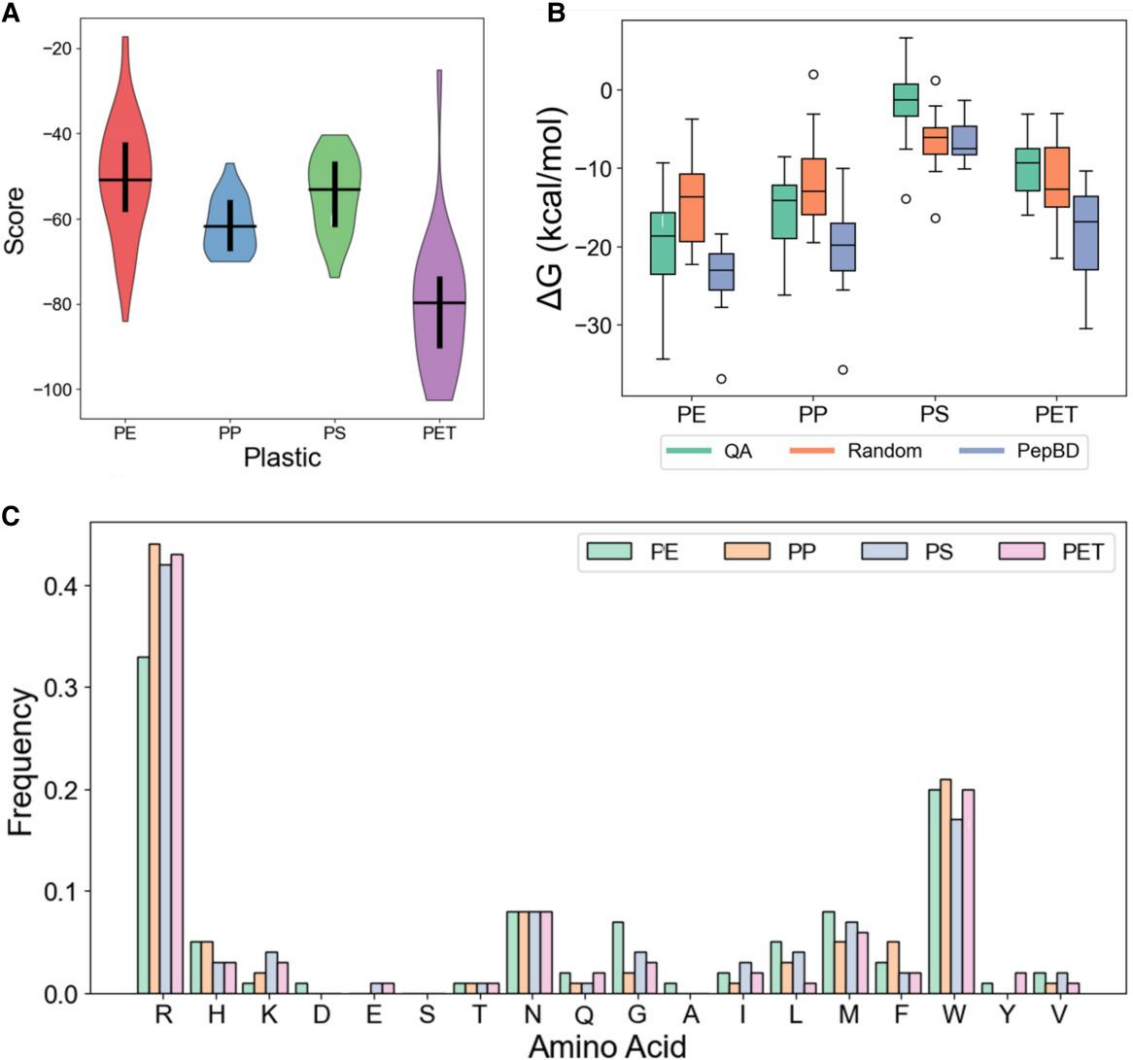
Properties of PBPs found by QA for four common plastics: PE, PS, PP, and PS. A) Range of scores for the best PBPs found by QA for PE, PP, PS, and PET. B) Distribution of adsorption free energies for PBPs to the four plastics in MD simulations. The PBPs were found either by QA, previously by PepBD, or by generating a random amino acid sequence. At least 12 PBPs were tested for each discovery method for each plastic. C) Amino acid frequency in the best PBPs found by QA for the four plastics. Data in (A) and (C) collected over 50 system conformations for PE and 24 system conformations for PP, PS, and PET.

Motivated by our success in finding PBPs for PE, we next searched for PBPs for other major components of MP waste: PP, PS, and PET. We found that the scores vary significantly between plastics (Fig. 3A). Designs for PET have the most negative scores (i.e. greatest predicted affinity), possibly because the oxygens in PET give rise to stronger electrostatic interactions with the peptide. The peptides for the other three plastics have roughly equivalent average scores, but the range of scores is much larger for PE than for PP or PS. While this could be attributable to the larger number of structures used for PE (53) than for the other plastics (24), the large range of scores for PET indicates that the plastic type also influences variability in the score magnitude. The peptides with the lowest scores for each plastic were evaluated in MD simulations and compared to previous PepBD peptides (49) along with randomly generated amino acid sequences (Fig. 3B). Twelve peptides were selected for each *method:plastic* combination so that a relatively large sample size could be evaluated at a reasonable computational cost. PBPs found by QA for PP had slightly lower affinity than previous PepBD peptides, but the best QA peptides have equal affinity to the best PepBD designs. In contrast, QA designs for PS and PET have lower affinity than both PepBD and random peptides. We suspect that this stems from deficiencies in the Potts model. The total two-body energy could dominate the one-body energy for some structures, largely due to the generalized Born (GB) solvation energy. This overemphasizes peptide stability relative to peptide–plastic interactions and leads to a high frequency of arginine (R) (Fig. 3C), whose long and flexible side chain forms strong intramolecular interactions with other peptide residues. Future work can explore striking a better balance of one- and two-body energies through tuning the value of *λ* for different plastics. Despite the difference in peptide affinity between plastics, the amino acid compositions are generally constant across all plastics (Fig. 3C). Notable exceptions are the slightly lower frequency of arginine (R) and the higher frequency of glycine (G) for PE and small variability in leucine (L) between the plastics. Statistical analysis using a two-tailed t test with unequal variance shows that these differences in amino acid frequencies are statistically significant at the *P* = 0.05 threshold (Table S1).

### Diversifying PBP physicochemical properties through PPO

**Fig. 4.**
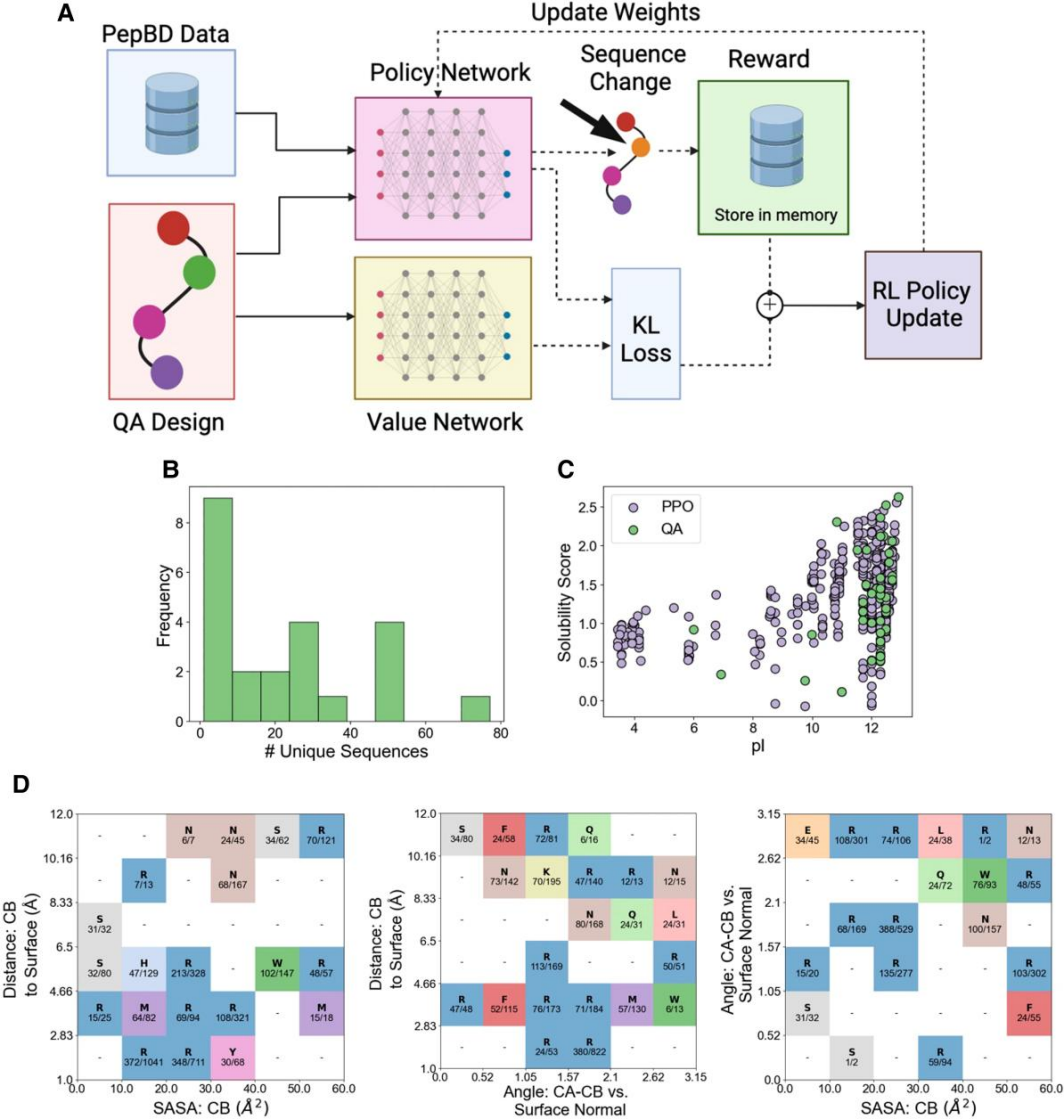
PPO diversifies PBP properties and provides insight into properties of optimal PBPs. A) Schematic of using PPO to identify additional PBPs. Details are provided in Materials and Methods. Figure created using Biorender. B) Number of alternate PBPs found by PPO for 23 PE system conformations. C) Net charge and solubility score found by QA or PPO. Each point corresponds to one peptide. D) The most frequently observed amino acids and counts of these amino acids in PBPs found for PE for a given geometric environment. Each cell contains the most frequently found amino acid and the number of times that that amino acid was observed. Results are only shown if an amino acid occurred in at least one-third of the sequences found by PPO. See text for definitions of the system conformations. Results taken from designs for 23 system conformations.

Having found PBPs with high predicted affinity for PE, we next aimed to find PBPs with a broader range of physicochemical properties using PPO. Diversity in physicochemical properties can be useful for MP remediation. The heterogeneity in both MP properties (e.g. surface charge) and environmental conditions (e.g. pH) (72) likely means that PBPs will not have high affinity for plastic in all settings. It thus could be helpful to have PBPs with diverse physicochemical properties so that a PBP can be selected for different settings as necessary. We selected PPO to search for alternate PBPs because it is effective at exploring high-dimensional spaces like amino acid sequences. In our PPO implementation (Fig. 4A), we trained PPO on the Potts model to learn a policy for exploring peptide sequences for a given system conformation. The trained PPO model searched for alternate solutions to the Potts model, starting with the sequence found by QA. A sampled sequence was deemed an alternate solution only if its score was within five units of the best score found by QA, thereby ensuring that the peptide was still predicted to have affinity for PE. PPO searched for alternate solutions over 23 system conformations, finding between 1 and 70 alternate amino acid sequences per system conformation (Fig. 4B). The number of alternate sequences found did not strongly correlate with the best score found by QA, i.e. the depth of the minima found by QA (Fig. S1). We next compared physicochemical properties of the QA and PPO peptides, namely the distribution of net charge, the predicted aqueous solubility (using the CamSol method (69)), the isoelectric point (pI) (using the ExPasy server(73)), and the peptide masses. While the two classes of peptides sample approximately the same range of these physiochemical properties, different combinations of the properties are sampled in PPO peptides sample than in QA peptides (Fig. S2). A particularly notable case is the combination of peptide solubility and pI (Fig. 4C). While nearly all QA peptides have a pI >10, a large fraction of PPO peptides have a pI below 7. As the MP surface charge can be either negative or positive (32), peptides with different net charges at neutral pH could aid MP remediation efforts. Overall, we conclude that PPO not only diversified peptide properties, but did so in a way that could have relevance to MP remediation (74).

PPO sampling provided insight into the relationship between the side-chain environment and the most optimal amino acid. The side-chain environment was characterized by three properties: (ⅰ) the distance between the beta carbon and the PE surface, (ⅱ) the angle between the vector connecting the alpha and beta carbons and the normal vector of the plastic surface, and (ⅲ) the solvent-accessible surface area (SASA) of the side-chain beta carbon when all other atoms in the corresponding side chain were removed. By calculating the side-chain environment of every residue in all PBPs found by PPO, the most frequent amino acid type was determined for each combination of geometric properties (Fig. 4D). An amino acid is shown only if a general preference was shown for that geometric environment, which we define as the amino acid occurring in more than one-third of all residues in that environment. Inspection of these plots reveals the most common amino acid for a given side-chain environment. Arginine (R) appears optimal when the side chain has a small SASA and is near the surface, or is distant from the surface and directed toward the solvent. Other hydrophilic residues like asparagine (N) and glutamine (Q) are also preferred when the residue is far from the plastic surface and exposed to the solvent. Meanwhile, bulky, hydrophobic residues like tryptophan (W), phenylalanine (F), methionine (M), and tyrosine (Y) are ideal when the side chain is near the surface and has a large SASA. This analysis could be helpful for peptide sequence optimization. While the 3D structure of the system predetermines the optimal amino acid sequence, it is not obvious what the optimal sequence is. The analysis in Fig. 4D simplifies this complicated design problem: the optimal amino acid sequence can be predicted by calculating the side-chain geometries, then selecting the best residue for that environment.

## Discussion

We developed a computational pipeline for discovering PBPs that could help remediate MP pollution. We created a biophysics-based Potts model that expresses peptide affinity for plastic as a function of the amino acid sequence for a given adsorbed conformation. The sequence with the highest predicted affinity was then found by QA for many different adsorbed conformations, giving a set of potential PBPs for multiple types of plastics commonly found in MP pollution. MD simulations showed that PBPs found by QA have high affinity for PE and PP. Application of PPO to the Potts model also increased diversity of the PBP physicochemical properties and shed light on the relationship between the local geometry of an amino acid side chain and the amino acid type predicted to be optimal.

The modeling and optimization approaches possess several desirable features. The outputs are explainable due to the biophysical foundation. Sequence optimization takes minutes, so large numbers of peptide conformations can be evaluated in the search for PBPs. As sampling of sequences and conformations are separate, the sequence optimization described in this work can be paired with a conformation sampling method to simultaneously search both spaces. This improves upon the current method of selecting conformations from MD simulations, which gives somewhat arbitrary conformations. Although QA was used to search for the optimal amino acid sequence, we recognize that not all researchers may have access to these resources. In such instances, QA could be replaced with classical optimization methods. Evaluation of PBPs via our MD protocol is high throughput: the results for nearly 150 peptides are shown in Fig. 3B. While the MD results are not high accuracy, it can serve as a useful first screen before more rigorous evaluations with methods like umbrella sampling (75, 76) or metadynamics (23, 24, 77). Computational modeling removes the need for experimental data that are required by many other AI-based optimization methods. This is useful given the lack of experimental data on PBPs. Lastly, the Potts model and MD simulations can quantitatively predict how peptide affinity is altered by changes in environmental conditions and MP properties. We thus can identify the conditions in which a PBP will most effectively remediate MP pollution, a direction that will be explored in future work.

The limitations of the modeling and optimization domains should also be noted. A primary limitation of the Potts model is the use of an implicit solvent model. While an implicit solvent model is necessary for peptide design to be tractable, the solvent plays an important role in peptide adsorption. A possible future option for including solvent effects would be to incorporate adsorption free energies of single amino acids taken from explicit solvent simulations (20) into the Potts model. As QA is a heuristics-based method, it may not find the global optimum. This issue is compounded by the proprietary D-Wave solvers, which may not be flexible enough to yield good solutions for all system conformations using the same parameters. Lastly, PPO has a large computational cost, may require tuning to the specific peptide design problem, and may require a good initial sequence to achieve optimal solutions. As PPO was used to tune the finding of peptides with unique properties, a possible solution for the issues with PPO is to instead perform QA design with modified versions of the Potts model that explicitly include terms for the property of interest.

**Fig. 5.**
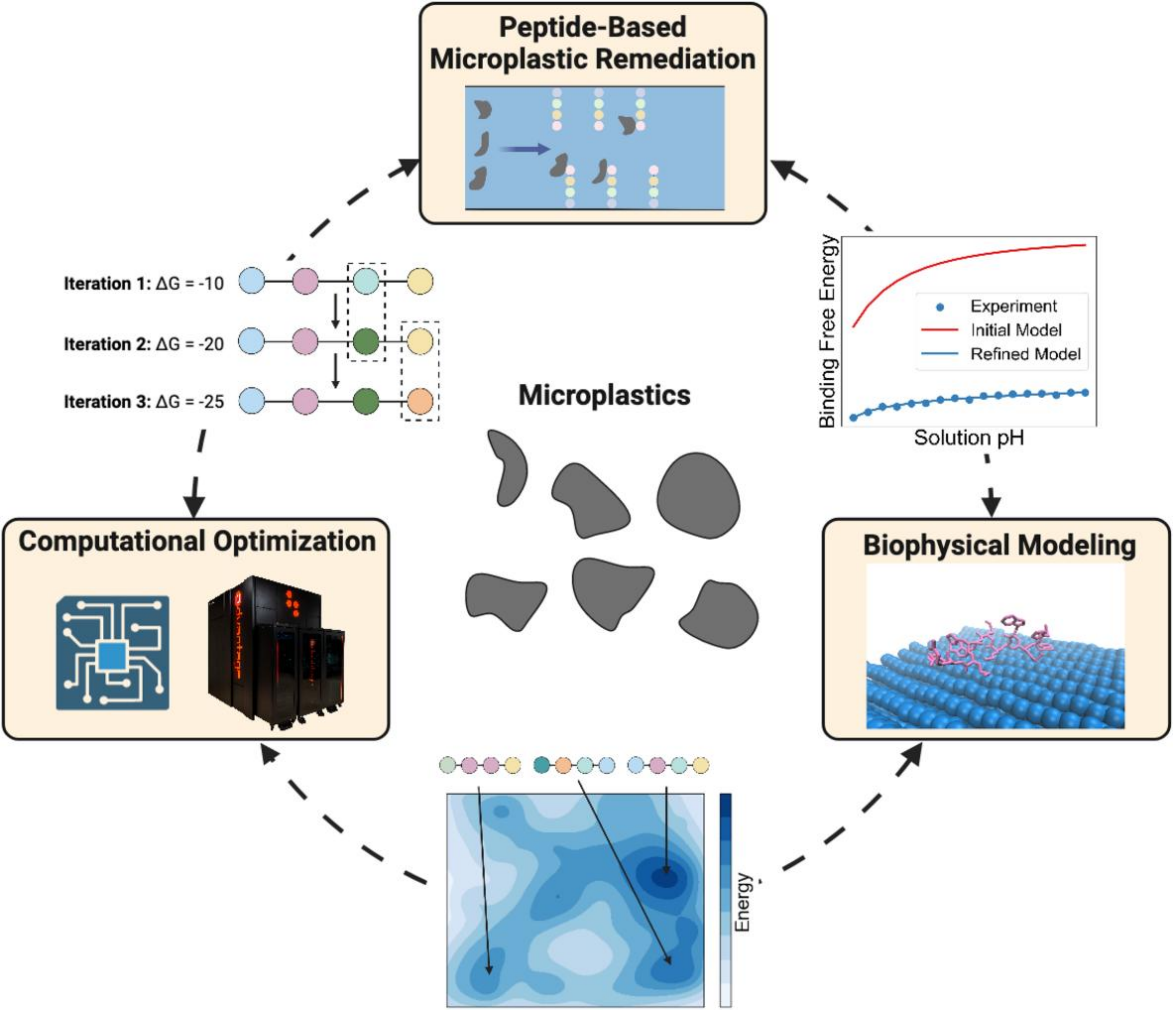
A nexus for developing peptide-based tools for MP remediation founded on biophysical modeling, computational optimization, and development of peptide-based MP remediation tools. See text for description of role of each domain, and how each domain interacts with the others. Figure created using Biorender.

This study sets the stage to develop peptide-based tools for remediating MP pollution, which we envision as the nexus shown in Fig. 5. To apply PBPs to MP pollution remediation, our computational methods must be paired with experimental work. A first essential step is to evaluate the computational predictions, namely the affinity of the peptides to plastic. Possible methods for performing this evaluation include quartz crystal microbalance (78) or atomic force microscopy (24). Since these methods can be time-consuming, a useful preliminary step would be to perform more rigorous computational evaluations using MD methods like metadynamics (79) or steered MD (80). The experimental and simulation results can provide feedback to the peptide design process, or even guide the search for higher affinity peptides by using methods like Gaussian processes or active learning. The second essential step is to incorporate PBPs into tools for MP remediation. Examples include creating biosensors for MP detection (30), using the peptides in wastewater treatment processes like bioflocculation to help capture MPs, and expressing the PBPs in plastic-degrading microorganisms (81) to aid cellular adhesion to plastic and accelerate plastic degradation. As these tools are developed, more information will be gained on peptide affinity to actual MPs in different environmental settings, thereby providing additional feedback to improve the computational peptide discovery method. The introduction of the computational and modeling domains in this work is a major step toward establishing a nexus for developing biological tools to remediate MP pollution.

## Materials and methods

### Expressing peptide sequence optimization as a Potts model

Because quantum annealers (QA) architecture are constrained to solving only discrete optimization problems, the peptide must be represented using discrete variables to enable the use of QA. Due to limited storage on QA machines, simultaneous representation of the peptide sequence and conformation (i.e. the backbone dihedrals and relative position and orientation of the peptide with respect to the plastic) is not possible. Thus, the aim is to optimize the peptide’s amino acid sequence for a fixed conformation. The quantity being optimized is the PepBD score, given by


(2) $$\mathrm{Score}=\;\Delta G_{\mathrm{MMGBSA}}+\lambda E_{\mathrm{pep}}$$

where $\Delta G_{\mathrm{MMGBSA}}$ is the binding free energy calculated using the MM/GBSA method (36), $E_{\mathrm{pep}}$ is the peptide internal energy that measures the stability of the peptide in the adsorbed state, and *λ* is a scalar that controls the relative importance of $E_{\mathrm{pep}}$ to $\Delta G_{\mathrm{MMGBSA}}$. To translate the PepBD score into the Potts model (69) format shown in Eq. (1), each peptide residue is assigned a state based on the type of amino acid present. All natural 20 amino acids are allowed except for proline. One-body energies are then the interactions of a given residue with the receptor and itself, while two-body energies are interactions between two residues. Just like in PepBD, intramolecular peptide interactions are scaled by a factor *λ* set to 0.01. For both one-body and two-body energies, the interaction energies consist of electrostatic, Lennard Jones, and GB energies between pairs of atoms. Evaluating the one- and two-body energies for all single amino acid and pairs of amino acids at all residues in the peptide, the net score for any amino acid sequence can be quickly determined by looking up the tabulated one- and two-body energies.

Two points in the Potts model merit discussion. The first point regards calculating Born radii (82) used in calculating the GB energy. PepBD uses the GB model of Onufriev (83), where the Born radii depend on the positions of all atoms in the system. This many-body calculation does not fit the Potts model. We resolved this issue by coarse graining all of the side chains that are not considered in the one- or two-body energy calculation. Coarse graining consisted of replacing the side chain with a sphere of radius 3 Ā placed at the beta carbon. The second point regards the side-chain orientations, or rotamers. While discretization of side-chain conformations using rotamer libraries (84) naturally fits a Potts model, the limited storage of QA machines prevents representation of both the amino acid sequence and rotamers. Thus, the side-chain degrees of freedom were removed. This was done by evaluating the one-body energy for all rotamers in a library (84) including a side-chain energy minimization step (85) and then only retaining the rotamer with the lowest one-body energy. We note that the retained rotamer could differ from residue to residue and that the two-body energy calculations used the single retained rotamer.

### Generating system configurations to formulate the Potts model

System conformations (i.e. peptide backbone dihedrals and the location and orientation of the peptide with respect to the plastic) were obtained in MD simulations. For PE, 20 system conformations were obtained using the method previously described (49) by running a metadynamics simulation with a bias potential applied to the distance between the peptide’s center of mass and the top of the surface. An additional 30 system conformations were obtained by randomly selecting 30 adsorbed conformations during evaluation of PepBD PBPs using the MD simulation procedure described below. The latter method was also used to obtain 24 system conformations for PP, PS, and PET.

### Discovering peptides by using QA to solve the Potts model

Quantum annealing was used to find the lowest scoring amino acid sequence for a given Potts model (see Fig. 1A). We used a D-Wave 2000Q QA with an annealing time of 120 μs and a chain strength of 8. Hyperparameters were optimized using a grid search approach to ensure efficient convergence.

The score function in Eq. 1 is slightly modified to include a constant penalty, *p,* to help QA escape local minima. The score function is given by


(3) $$\mathrm{Score}\;=\;\underset{i}{\sum }\;E_{i}s_{i}+\lambda \underset{i}{\sum }\;\underset{\;j>i}{\sum }\;E_{ij}s_{i}s_{j}+p$$

A variety of *λ* and *p* values are used for each system conformation to obtain multiple solutions for each Potts model. The Potts model was solved with the Kerberos D-Wave hybrid solver (86), which combines quantum and classical algorithms to balance the ability of quantum computing to find local minima with the scalability of classical computing. Namely, the D-Wave hybrid solver Kerberos decomposes the Potts model into subproblems that are solved with the QA, while the classical algorithm refines and integrates QA solutions to the subproblems.

### Evaluation of peptides via MD simulations

PBPs affinities to plastic were evaluated in MD simulations. Our need for evaluating dozens of peptides required a high-throughput, computationally cheap method. This rules out accurate but expensive methods like metadynamics (23) or umbrella sampling (87). We instead run equilibrium MD simulations to search for the most stable adsorbed conformation, measuring affinities using MM/GBSA. As peptide conformations change slowly when adsorbed to a plastic at 300 K, we first simulated the peptide for 10-ns simulation at 550 K. The elevated temperature allowed the peptide to rapidly sample different adsorbed conformations. The Upper Wall utility in PLUMED (88) was used to prevent the peptide from desorbing completely from the plastic surface, specifically by applying a force to push the peptide back toward the plastic if its center of mass was more than 10 Å away from the plastic. Sixteen representative adsorbed conformations for each peptide were then obtained by first performing *k*-means clustering with CPPTRAJ (89) based on the backbone alpha carbons and then randomly selecting a member of each cluster. Each conformation was simulated for 1 ns equilibrated at 300 K before calculating the MM/GBSA adsorption free energy with Amber (90) to identify the most stable conformations. The eight conformations with the lowest adsorption free energy were simulated an additional 4 ns before repeating the adsorption free energy calculation. The lowest adsorption free energy was selected as representative of the peptide’s binding affinity.

Technical details of MD simulations are the following. Simulations used TIP3P water (91) and the ff14SB force field (92) for peptides. Plastics were modeled using GAFF (93) parameters and previously calculated partial charges (49). Atomistic models of plastic surfaces were taken from the same work (49). tLEaP (94) generated a linear peptide with the desired amino acid sequence, which was manually translated above the plastic surface using VMD (95) so that its long dimension was parallel to the surface and the peptide center of mass 4 Å was above the surface. tLEaP added TIP3P water 15 Å above the peptide and 10 Å below the bottom of the plastic surface, giving simulation box sizes of about 50 Å in the direction normal to the plastic surface. The dimensions of the periodic box parallel to the plastic surface were set to be equal to the dimensions of the plastic surface. The Amber coordinate and parameter files were converted to Gromacs format using Parmed (94) before running simulations with Gromacs version 2019.6 (96). Position restraints were applied to nonhydrogens in the plastic using a force constant of 5,000 kJ/mol/nm^2^. All bonds to hydrogen were restrained using the LINCS algorithm (97). Prior to running a high-temperature 550-K simulation, the system was energy-minimized for up to 1,000 steps using steepest descent, equilibrated at 300 K in the NVT ensemble for 100 ps, and equilibrated in the NPT ensemble system at 1 bar and 300 K for 200 ps. The system was then equilibrated at 550 K for 200 ps in the NVT ensemble before running the 10-ns high-temperature simulation. After extracting clusters, the system was cooled to 300 K in the NVT ensemble for 100 ps before running the 1- and 4-ns simulations of each cluster. Long-range electrostatic interactions were treated using particle mesh Ewald. The simulation time step size was 2 fs. The velocity rescaling algorithm (98) controlled the system temperature in NVT and NPT. The time constant was 0.1 ps, and separate thermostats were applied to water and nonwater atoms (i.e. the peptide, the plastic, and any counterions). The semi-isotropic Berendsen barostat (99) controlled the pressure in NPT simulations. The *x* and *y* dimensions were allowed to change independently from the *z* dimension, the isothermal compressibility was set to 4.5 × 10^−4^ for all directions, and the time constant was set to 5 ps.

### Expanding the biophysical properties of PBPs with PPO

Proximal policy optimization, a prominent reinforcement learning algorithm, can stably and efficiently navigate complex environments. Classic PPO methods employs a clipping function within its objective that prevents unstable policy updates, thereby ensuring that updated policies remain close to their predecessors. An example of a learning curve for PPO is shown in Fig. S3. We note that QA still plays a key role in PBP discovery: using the QA sequence as the starting point for PPO exploration greatly accelerates discovery of alternate solutions with good scores (Fig. S4). This process maintains equilibrium between exploring updated strategies and exploiting known, effective behaviors. PPO’s strategy, including actions guided by current policies and evaluated via a reward system, alongside controlled policy modifications through KL divergence, underscores its robustness in complex optimization tasks.

Our PPO neural network comprises an embedding layer and a gated recurrent unit (GRU) that processes sequential amino acid data (Fig. S5). The embedding layer translates the discrete amino acid inputs into a continuous vector space, enhancing the network’s ability to discern patterns in peptide sequences. The GRU can capture how the individual amino acids interact to give the score of the full amino acid sequence. The output layer, which is connected to the GRU, employs a softmax function to generate a probability distribution over potential actions (e.g. changing an amino acid). This setup allows the policy network to probabilistically determine the next amino acid in a sequence, facilitating exploration of the vast peptide sequence space with the aim of optimizing the peptide score. Details of the method follow below.

#### Embedding layer

This layer maps discrete amino acid inputs into a continuous vector space. Mathematically, each amino acid $AA_{i}$ is transformed into an embedded vector $v_{i}$ through an embedding function $E:\;AA_{i}\to v_{i}$. This transformation enhances the network’s ability to identify intricate patterns in peptide sequences.

#### GRU layer

The GRU is adept at processing sequential data and capturing temporal dependencies. For a given state $s_{t}$ (defined below), the GRU updates its hidden state $h_{t}$ at each time step *t* per $h_{t}=\mathrm{GRU}(s_{t},h_{t-1})$. This mechanism allows the network to maintain a memory of previous amino acids in the sequence, which is crucial for predicting subsequent amino acids.

#### Output layer with softmax activation

Connected to the GRU, the output layer utilizes a softmax function to generate a probability distribution of possible actions (amino acid selections):


(4) $$P(a_{t}|s_{t};\theta )=\mathrm{softmax}(W_{\mathrm{out}}h_{t}+b_{\mathrm{out}})$$

where $W_{\mathrm{out}}$ and $b_{\mathrm{out}}$ are the weights and biases of the output layer, respectively. This probabilistic approach enables the policy network to determine the next amino acid in the sequence, thus systematically exploring the peptide sequence space.

The definition of states, actions, and rewards is intricately tied to the task of optimizing peptide sequences. The state is the current sequence of amino acids, the action is a change in the amino acid at a specific position in the peptide, and the reward is the change in the score after modifying the amino acid sequence. PPO aims to learn a policy that goes toward lower scores, which corresponds to peptides with higher predicted affinity to the plastic. We define these components as follows:

**States (S)**: The state $s_{t}$ is the current amino acid sequence:


(5) $$s_{t}=\{AA_{1},\ldots ,AA_{n}\}$$

where $AA_{i}$ denotes the amino acid at position *i* in the peptide chain and *n* is the length of the peptide sequence.

**Actions (A)**: An action is the selection and insertion of an amino acid at a specific position within the peptide sequence.


(6) $$a_{t}:s_{t}\to s_{t+1}$$

where $s_{t+1}$ is the updated state after the action is applied.

**Rewards (R)**: The reward $R(s_{t},a_{t})$ is defined to be the change in the score for the peptide sequence from before to after the action $a_{t}$:


(7) $$R(s_{t},a_{t})=\mathrm{QUBO}(s_{t+1})-\mathrm{QUBO}(s_{t})$$

This reward encourages the policy to sample peptide sequences with high predicted affinity.

## Supplementary Material

pgae572_Supplementary_Data

## Data Availability

The code for the Potts Model can be found at https://github.com/CarolHall-NCSU-CBE/PepBD_PottsModel. The code for running QA and PPO and all data can be found at https://github.com/PEESEgroup/QA-PBP-RL-Nexus.

## References

[1] Cox KD , et al 2019. Human consumption of microplastics. Environ Sci Technol. 53:7068–7074. 31184127 10.1021/acs.est.9b01517

[2] Vethaak AD , Legler J. 2021. Microplastics and human health. Science. 371:672–674. 33574197 10.1126/science.abe5041

[3] Zhao X , You F. 2022. Life cycle assessment of microplastics reveals their greater environmental hazards than mismanaged polymer waste losses. Environ Sci Technol. 56:11780–11797. 35920730 10.1021/acs.est.2c01549

[4] Zhao B , Richardson RE, You F. 2024. Microplastics monitoring in freshwater systems: a review of global efforts, knowledge gaps, and research priorities. J Hazard Mater. 477:135329. 39088945 10.1016/j.jhazmat.2024.135329

[5] Zhao X , You F. 2024. Microplastic human dietary uptake from 1990 to 2018 grew across 109 major developing and industrialized countries but can be halved by plastic debris removal. Environ Sci Technol. 58:8709–8723. 38656828 10.1021/acs.est.4c00010 PMC11112738

[6] Mukherjee F , Shi A, Wang X, You F, Abbott NL. 2023. Liquid crystals as multifunctional interfaces for trapping and characterizing colloidal microplastics. Small. 19:2207802. 10.1002/smll.202207802 36892170

[7] Bang RS , Verster L, Hong H, Pal L, Velev OD. 2024. Colloidal engineering of microplastic capture with biodegradable soft dendritic “microcleaners.”. Langmuir. 40:5923–5933. 38428025 10.1021/acs.langmuir.3c03869

[8] Li J , Liu H, Paul Chen J. 2018. Microplastics in freshwater systems: a review on occurrence, environmental effects, and methods for microplastics detection. Water Res. 137:362–374. 29580559 10.1016/j.watres.2017.12.056

[9] Sgier L , Freimann R, Zupanic A, Kroll A. 2016. Flow cytometry combined with viSNE for the analysis of microbial biofilms and detection of microplastics. Nat Commun. 7:11587. 27188265 10.1038/ncomms11587 PMC4873979

[10] Zhao X , You F. 2024. From sustainable macro debris chemical recycling to microplastic reclamation: overview, research challenges, and outlook. J Clean Prod. 454:142281.

[11] Adamczyk Z . 2019. Protein adsorption: a quest for a universal mechanism. Curr Opin Colloid Interface Sci. 41:50–65.

[12] Browne MM , Lubarsky GV, Davidson MR, Bradley RH. 2004. Protein adsorption onto polystyrene surfaces studied by XPS and AFM. Surf Sci. 553:155–167.

[13] Firkowska-Boden I , Zhang X, Jandt KD. 2018. Controlling protein adsorption through nanostructured polymeric surfaces. Adv Healthc Mater. 7:1700995. 10.1002/adhm.201700995 29193909

[14] Kumar N , Parajuli O, Gupta A, Hahm J. 2008. Elucidation of protein adsorption behavior on polymeric surfaces: toward high-density, high-payload protein templates. Langmuir. 24:2688–2694. 18225924 10.1021/la7022456

[15] Rabe M , Verdes D, Seeger S. 2011. Understanding protein adsorption phenomena at solid surfaces. Adv Colloid Interface Sci. 162:87–106. 21295764 10.1016/j.cis.2010.12.007

[16] Vogler EA . 2012. Protein adsorption in three dimensions. Biomaterials. 33:1201–1237. 22088888 10.1016/j.biomaterials.2011.10.059 PMC3278642

[17] Zhao W , Xu Z, Cui Q, Sahai N. 2016. Predicting the structure–activity relationship of hydroxyapatite-binding peptides by enhanced-sampling molecular simulation. Langmuir. 32:7009–7022. 27329793 10.1021/acs.langmuir.6b01582

[18] Canabady-Rochelle LLS , et al 2012. Bioinspired silicification of silica-binding peptide-silk protein chimeras: comparison of chemically and genetically produced proteins. Biomacromolecules. 13:683–690. 22229696 10.1021/bm201555c PMC3304446

[19] Hughes ZE , Walsh TR. 2015. What makes a good graphene-binding peptide? Adsorption of amino acids and peptides at aqueous graphene interfaces. J Mater Chem B. 3:3211–3221. 32262315 10.1039/c5tb00004a

[20] Hughes ZE , et al 2021. Tuning materials-binding peptide sequences toward gold- and silver-binding selectivity with Bayesian optimization. ACS Nano. 15:18260–18269. 34747170 10.1021/acsnano.1c07298

[21] Care A , Bergquist PL, Sunna A. 2015. Solid-binding peptides: smart tools for nanobiotechnology. Trends Biotechnol. 33:259–268. 25796487 10.1016/j.tibtech.2015.02.005

[22] Qi X , Pfaendtner J. 2024. High-throughput computational screening of solid-binding peptides. J Chem Theory Comput. 20:2959–2968. 38499981 10.1021/acs.jctc.3c01286

[23] Deighan M , Pfaendtner J. 2013. Exhaustively sampling peptide adsorption with metadynamics. Langmuir. 29:7999–8009. 23706011 10.1021/la4010664

[24] Horst Meißner R , Wei G, Colombi Ciacchi L. 2015. Estimation of the free energy of adsorption of a polypeptide on amorphous SiO 2 from molecular dynamics simulations and force spectroscopy experiments. Soft Matter. 11:6254–6265. 26158561 10.1039/c5sm01444a

[25] Palafox-Hernandez JP , et al 2014. Comparative study of materials-binding peptide interactions with gold and silver surfaces and nanostructures: a thermodynamic basis for biological selectivity of inorganic materials. Chem Mater. 26:4960–4969.

[26] Casals E , Pfaller T, Duschl A, Oostingh GJ, Puntes V. 2010. Time evolution of the nanoparticle protein Corona. ACS Nano. 4:3623–3632. 20553005 10.1021/nn901372t

[27] Gopinath PM , et al 2019. Assessment on interactive prospectives of nanoplastics with plasma proteins and the toxicological impacts of virgin, coronated and environmentally released-nanoplastics. Sci Rep. 9:8860. 31222081 10.1038/s41598-019-45139-6 PMC6586940

[28] Lundqvist M , et al 2008. Nanoparticle size and surface properties determine the protein corona with possible implications for biological impacts. Proc Natl Acad Sci U S A. 105:14265–14270. 18809927 10.1073/pnas.0805135105 PMC2567179

[29] Ke PC , Lin S, Parak WJ, Davis TP, Caruso F. 2017. A decade of the protein corona. ACS Nano. 11:11773–11776. 29206030 10.1021/acsnano.7b08008

[30] Oh S , et al 2021. Peptide specific nanoplastic detection based on sandwich typed localized surface plasmon resonance. Nanomaterials. 11:2887. 34835653 10.3390/nano11112887 PMC8617854

[31] Woo H , et al 2022. Sensitive and specific capture of polystyrene and polypropylene microplastics using engineered peptide biosensors. RSC Adv. 12:7680–7688. 35424716 10.1039/d1ra08701k PMC8982333

[32] Bakhshinejad B , Sadeghizadeh M. 2016. A polystyrene binding target-unrelated peptide isolated in the screening of phage display library. Anal Biochem. 512:120–128. 27555439 10.1016/j.ab.2016.08.013

[33] Qiang X , et al 2017. Discovery of a polystyrene binding peptide isolated from phage display library and its application in peptide immobilization. Sci Rep. 7:2673. 28572662 10.1038/s41598-017-02891-x PMC5453990

[34] Vodnik M , Štrukelj B, Lunder M. 2012. HWGMWSY, an unanticipated polystyrene binding peptide from random phage display libraries. Anal Biochem. 424:83–86. 22370277 10.1016/j.ab.2012.02.013

[35] Serizawa T , Techawanitchai P, Matsuno H. 2007. Isolation of peptides that can recognize syndiotactic polystyrene. ChemBioChem. 8:989–993. 17492699 10.1002/cbic.200700133

[36] Wang E , et al 2019. End-point binding free energy calculation with MM/PBSA and MM/GBSA: strategies and applications in drug design. Chem Rev. 119:9478–9508. 31244000 10.1021/acs.chemrev.9b00055

[37] Raut VP , Agashe MA, Stuart SJ, Latour RA. 2005. Molecular dynamics simulations of peptide−surface interactions. Langmuir. 21:1629–1639. 15697318 10.1021/la047807f

[38] Corni S , Hnilova M, Tamerler C, Sarikaya M. 2013. Conformational behavior of genetically-engineered dodecapeptides as a determinant of binding affinity for gold. J. Phys. Chem. C. 117:16990–17003.

[39] Budi A , Walsh TR. 2019. A bespoke force field to describe biomolecule adsorption at the aqueous boron nitride interface. Langmuir. 35:16234–16243. 31714785 10.1021/acs.langmuir.9b03121

[40] Edwards RJ , et al 2007. Bioinformatic discovery of novel bioactive peptides. Nat Chem Biol. 3:108–112. 17220901 10.1038/nchembio854

[41] Oren EE , et al 2007. A novel knowledge-based approach to design inorganic-binding peptides. Bioinformatics. 23:2816–2822. 17875545 10.1093/bioinformatics/btm436

[42] Parvizpour S , Pourseif MM, Razmara J, Rafi MA, Omidi Y. 2020. Epitope-based vaccine design: a comprehensive overview of bioinformatics approaches. Drug Discov Today. 25:1034–1042. 32205198 10.1016/j.drudis.2020.03.006

[43] van Laarhoven PJM , Aarts EHL. “Simulated annealing”. In: van Laarhoven PJM, Aarts EHL, editors. Simulated annealing: theory and applications, mathematics and its applications. Springer Netherlands, 1987. p. 7–15.

[44] Bertsimas D , Tsitsiklis J. 1993. Simulated annealing. Stat Sci. 8:10–15.

[45] Holland JH . 1992. Genetic algorithms. Sci Am. 267:66–73.1411454

[46] Schmitt LM . 2001. Theory of genetic algorithms. Theor Comput Sci. 259:1–61.

[47] Hu X , et al 2020. Recent advances in short peptide self-assembly: from rational design to novel applications. Curr Opin Colloid Interface Sci. 45:1–13.

[48] Lins L , Charloteaux B, Heinen C, Thomas A, Brasseur R. 2006. “De novo” design of peptides with specific lipid-binding properties. Biophys J. 90:470–479. 16275638 10.1529/biophysj.105.068213 PMC1367053

[49] Bergman MT , Xiao X, Hall CK. 2023. In silico design and analysis of plastic-binding peptides. J Phys Chem B. 127:8370–8381. 37735840 10.1021/acs.jpcb.3c04319 PMC10591858

[50] Manavalan B , Subramaniyam S, Shin TH, Kim MO, Lee G. 2018. Machine-learning-based prediction of cell-penetrating peptides and their uptake efficiency with improved accuracy. J Proteome Res. 17:2715–2726. 29893128 10.1021/acs.jproteome.8b00148

[51] Batra R , et al 2022. Machine learning overcomes human bias in the discovery of self-assembling peptides. Nat Chem. 14:1427–1435. 36316409 10.1038/s41557-022-01055-3 PMC9844539

[52] Wang J , et al 2023. Deep learning empowers the discovery of self-assembling peptides with over 10 trillion sequences. Advanced Science. 10:2301544. 37749875 10.1002/advs.202301544 PMC10625107

[53] Guntuboina C , Das A, Mollaei P, Kim S, Barati Farimani A. 2023. PeptideBERT: a language model based on transformers for peptide property prediction. J Phys Chem Lett. 14:10427–10434. 37956397 10.1021/acs.jpclett.3c02398 PMC10683064

[54] S. Alamdari , et al 12 September 2023. Protein generation with evolutionary diffusion: sequence is all you need. bioRxiv 2023.09.11.556673. 10.1101/2023.09.11.556673, preprint: not peer reviewed.

[55] Ingraham JB , et al 2023. Illuminating protein space with a programmable generative model. Nature. 623:1070–1078. 37968394 10.1038/s41586-023-06728-8 PMC10686827

[56] Watson JL , et al 2023. De novo design of protein structure and function with RFdiffusion. Nature. 620:1089–1100. 37433327 10.1038/s41586-023-06415-8 PMC10468394

[57] Das P , et al 2021. Accelerated antimicrobial discovery via deep generative models and molecular dynamics simulations. Nat Biomed Eng. 5:613–623. 33707779 10.1038/s41551-021-00689-x

[58] Nagarajan D , et al 2018. Computational antimicrobial peptide design and evaluation against multidrug-resistant clinical isolates of bacteria. J Biol Chem. 293:3492–3509. 29259134 10.1074/jbc.M117.805499 PMC5846155

[59] Yu H , Wang R, Qiao J, Wei L. 2024. Multi-CGAN: deep generative model-based multiproperty antimicrobial peptide design. J Chem Inf Model. 64:316–326. 38135439 10.1021/acs.jcim.3c01881

[60] Wang C , Garlick S, Zloh M. 2021. Deep learning for novel antimicrobial peptide design. Biomolecules. 11:471. 33810011 10.3390/biom11030471 PMC8004669

[61] Yu L , Jing R, Liu F, Luo J, Li Y. 2020. DeepACP: a novel computational approach for accurate identification of anticancer peptides by deep learning algorithm. Mol Ther Nucleic Acids. 22:862–870. 33230481 10.1016/j.omtn.2020.10.005 PMC7658571

[62] Grisoni F , et al 2018. Designing anticancer peptides by constructive machine learning. ChemMedChem. 13:1300–1302. 29679519 10.1002/cmdc.201800204

[63] Grisoni F , et al 2019. De novo design of anticancer peptides by ensemble artificial neural networks. J Mol Model. 25:112. 30953170 10.1007/s00894-019-4007-6

[64] Chen J , Cheong HH, Siu SWI. 2021. xDeep-AcPEP: deep learning method for anticancer peptide activity prediction based on convolutional neural network and multitask learning. J Chem Inf Model. 61:3789–3803. 34327990 10.1021/acs.jcim.1c00181

[65] de Oliveira ECL , Santana K, Josino L, Lima e Lima AH, de Souza de Sales Júnior C. 2021. Predicting cell-penetrating peptides using machine learning algorithms and navigating in their chemical space. Sci Rep. 11:7628. 33828175 10.1038/s41598-021-87134-w PMC8027643

[66] Cao Y , Romero J, Aspuru-Guzik A. 2018. Potential of quantum computing for drug discovery. IBM J Res Dev. 62:6:1–6:20.

[67] V. K. Mulligan , et al 2 September 2019. Designing peptides on a quantum computer. bioRxiv 752485. 10.1101/752485, preprint: not peer reviewed.

[68] Tučs A , et al 2023. Quantum annealing designs nonhemolytic antimicrobial peptides in a discrete latent space. ACS Med Chem Lett. 14:577–582. 37197452 10.1021/acsmedchemlett.2c00487 PMC10184305

[69] Wu FY . 1982. The Potts model. Rev Mod Phys. 54:235–268.

[70] Xiao X , Agris PF, Hall CK. 2015. Designing peptide sequences in flexible chain conformations to bind RNA: a search algorithm combining Monte Carlo, self-consistent mean field and concerted rotation techniques. J Chem Theory Comput. 11:740–752. 26579605 10.1021/ct5008247

[71] Xiao X , Wang Y, Leonard JN, Hall CK. 2017. Extended concerted rotation technique enhances the sampling efficiency of the computational peptide-design algorithm. J Chem Theory Comput. 13:5709–5720. 29023116 10.1021/acs.jctc.7b00714 PMC12928211

[72] Bang RS , et al 2023. An integrated chemical engineering approach to understanding microplastics. AIChE J. 69:e18020.

[73] Gasteiger E , et al 2003. ExPASy: the proteomics server for in-depth protein knowledge and analysis. Nucleic Acids Res. 31:3784–3788. 12824418 10.1093/nar/gkg563 PMC168970

[74] Sormanni P , Aprile FA, Vendruscolo M. 2015. The CamSol method of rational design of protein mutants with enhanced solubility. J Mol Biol. 427:478–490. 25451785 10.1016/j.jmb.2014.09.026

[75] Degen GD , Cunha KC, Levine ZA, Waite JH, Shea J-E. 2021. Molecular context of Dopa influences adhesion of mussel-inspired peptides. J Phys Chem B. 125:9999–10008. 34459591 10.1021/acs.jpcb.1c05218

[76] O’Brien CP , Stuart SJ, Bruce DA, Latour RA. 2008. Modeling of peptide adsorption interactions with a poly(lactic acid) surface. Langmuir. 24:14115–14124. 19360943 10.1021/la802588n PMC2771889

[77] Mao CM , Sampath J, Sprenger KG, Drobny G, Pfaendtner J. 2019. Molecular driving forces in peptide adsorption to metal oxide surfaces. Langmuir. 35:5911–5920. 30955325 10.1021/acs.langmuir.8b01392

[78] Mermut O , et al 2006. In situ adsorption studies of a 14-amino acid leucine-lysine peptide onto hydrophobic polystyrene and hydrophilic silica surfaces using quartz crystal microbalance, atomic force microscopy, and sum frequency generation vibrational spectroscopy. J Am Chem Soc. 128:3598–3607. 16536533 10.1021/ja056031h

[79] Bussi G , Laio A. 2020. Using metadynamics to explore complex free-energy landscapes. Nat Rev Phys. 2:200–212.

[80] Park S , Schulten K. 2004. Calculating potentials of mean force from steered molecular dynamics simulations. J Chem Phys. 120:5946–5961. 15267476 10.1063/1.1651473

[81] Li T , Menegatti S, Crook N. 2023. Breakdown of polyethylene therepthalate microplastics under saltwater conditions using engineered Vibrio natriegens. AIChE J. 69:e18228.

[82] Onufriev A , Case DA, Bashford D. 2002. Effective Born radii in the generalized Born approximation: the importance of being perfect. J Comput Chem. 23:1297–1304. 12214312 10.1002/jcc.10126

[83] Onufriev A , Bashford D, Case DA. 2004. Exploring protein native states and large-scale conformational changes with a modified generalized born model. Proteins: Struct, Funct, Bioinf. 55:383–394. 10.1002/prot.20033 15048829

[84] Lovell SC , Word JM, Richardson JS, Richardson DC. 2000. The penultimate rotamer library. Proteins: Struct, Funct, Bioinf. 40:389–408. 10861930

[85] Xiao X , Hung ME, Leonard JN, Hall CK. 2016. Adding energy minimization strategy to peptide-design algorithm enables better search for RNA-binding peptides: redesigned λ N peptide binds boxB RNA. J Comput Chem. 37:2423–2435. 27487990 10.1002/jcc.24466 PMC5314887

[86] D-Wave Systems . D-Wave Ocean Software Documentation—Ocean Documentation 6.9.0 documentation. [Accessed 2024 April 14]. https://docs.ocean.dwavesys.com/en/stable/.

[87] Levine ZA , et al 2016. Surface force measurements and simulations of mussel-derived peptide adhesives on wet organic surfaces. Proc Natl Acad Sci U S A. 113:4332–4337. 27036002 10.1073/pnas.1603065113 PMC4843488

[88] Tribello GA , Bonomi M, Branduardi D, Camilloni C, Bussi G. 2014. PLUMED 2: new feathers for an old bird. Comput Phys Commun. 185:604–613.

[89] Roe DR , Cheatham TE. 2013. PTRAJ and CPPTRAJ: software for processing and analysis of molecular dynamics trajectory data. J Chem Theory Comput. 9:3084–3095. 26583988 10.1021/ct400341p

[90] Case DA , et al 2023. AmberTools. J Chem Inf Model. 63:6183–6191. 37805934 10.1021/acs.jcim.3c01153 PMC10598796

[91] Jorgensen WL . 1981. Quantum and statistical mechanical studies of liquids. 10. Transferable intermolecular potential functions for water, alcohols, and ethers. Application to liquid water. J Am Chem Soc. 103:335–340.

[92] Maier JA , et al 2015. ff14SB: improving the accuracy of protein side chain and backbone parameters from ff99SB. J Chem Theory Comput. 11:3696–3713. 26574453 10.1021/acs.jctc.5b00255 PMC4821407

[93] Vassetti D , Pagliai M, Procacci P. 2019. Assessment of GAFF2 and OPLS-AA general force fields in combination with the water models TIP3P, SPCE, and OPC3 for the solvation free energy of druglike organic molecules. J Chem Theory Comput. 5:1983–1995. 10.1021/acs.jctc.8b01039 30694667

[94] CASE DA , et al 2005. The amber biomolecular simulation programs. J Comput Chem. 26:1668–1688. 16200636 10.1002/jcc.20290 PMC1989667

[95] Humphrey W , Dalke A, Schulten K. 1996. VMD: visual molecular dynamics. J Mol Graph. 14:33–38. 8744570 10.1016/0263-7855(96)00018-5

[96] Abraham MJ , et al 2015. GROMACS: high performance molecular simulations through multi-level parallelism from laptops to supercomputers. SoftwareX. 1–2:19–25.

[97] Hess B , Bekker H, Berendsen HJC, Fraaije JGEM. 1997. LINCS: a linear constraint solver for molecular simulations. J Comput Chem. 18:1463–1472.

[98] Bussi G , Donadio D, Parrinello M. 2007. Canonical sampling through velocity rescaling. J Chem Phys. 126:014101. 17212484 10.1063/1.2408420

[99] Berendsen HJC , Postma JPM, van Gunsteren WF, DiNola A, Haak JR. 1984. Molecular dynamics with coupling to an external bath. J Chem Phys. 81:3684–3690.

