# Supplementary material for: Integrating biophysical modeling, quantum computing, and AI to discover plastic-binding peptides that combat microplastic pollution
Source: PNAS Nexus. 2025 Feb 18;4(2):pgae572. doi: 10.1093/pnasnexus/pgae572 (PMC11770337; doi:10.1093/pnasnexus/pgae572)
Supplement: pgae572_Supplementary_Data [file pgae572_supplementary_data.zip › PeptideDesign_SI_PNAS_Nexus.docx]

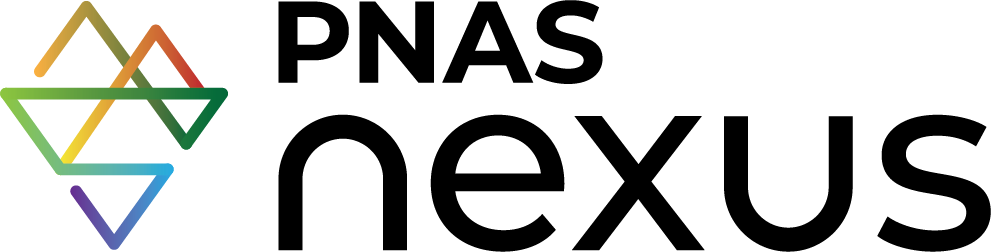


**Supplementary Information for**

**Integrating biophysical modeling, quantum computing, and AI to discover plastic-binding peptides that combat microplastic pollution**

Jeet Dhoriyani^1#^, Michael T. Bergman^2#^, Carol K. Hall^2*^, and Fengqi You^1,3,4*^

^1^Systems Engineering, College of Engineering, Cornell University, Ithaca, New York 14853, USA

^2^Department of Chemical and Biomolecular Engineering, North Carolina State University, Raleigh, NC 27606, USA

^3^Robert Frederick Smith School of Chemical and Biomolecular Engineering, Cornell University, Ithaca, New York 14853, USA

^4^Cornell University AI for Science Institute, Cornell University, Ithaca, New York 14853, USA

^#^These authors contributed equally to this work

* To whom correspondence should be addressed

**Emails:**  hall@ncsu.edu fengqi.you@cornell.edu

**This PDF file includes:**

Figure S1-S5

Table S1

**Other supporting materials for this manuscript include the following:**

Dataset S1


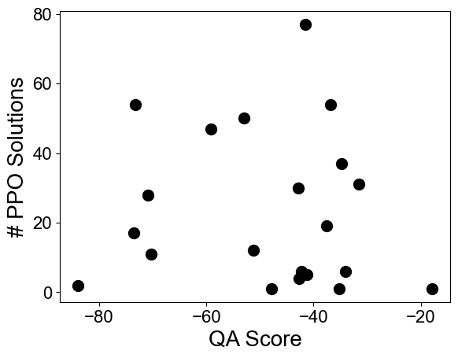


**Figure S1. Number of solutions found by PPO as a function of the best score found by QA for the system conformation.** Results are shown for 23 system conformations used by PPO to design peptides that bind to polyethylene.

Figure S1 shows the correlation between the number of peptides discovered by proximal policy optimization (PPO) for a given system conformation, and the best (i.e. lowest) scoring peptide found by quantum annealing (QA). The score found by QA is either equal to or a good approximation of the global minimum of the Potts model formulated for the system conformation. Peptides sampled by PPO were only counted if their score was within 5 of the best score found by QA. No strong correlation is found between the QA score and the number of peptides discovered by PPO. It thus appears that the number of alternate solutions is not strongly dependent on the depth of the global minimum.


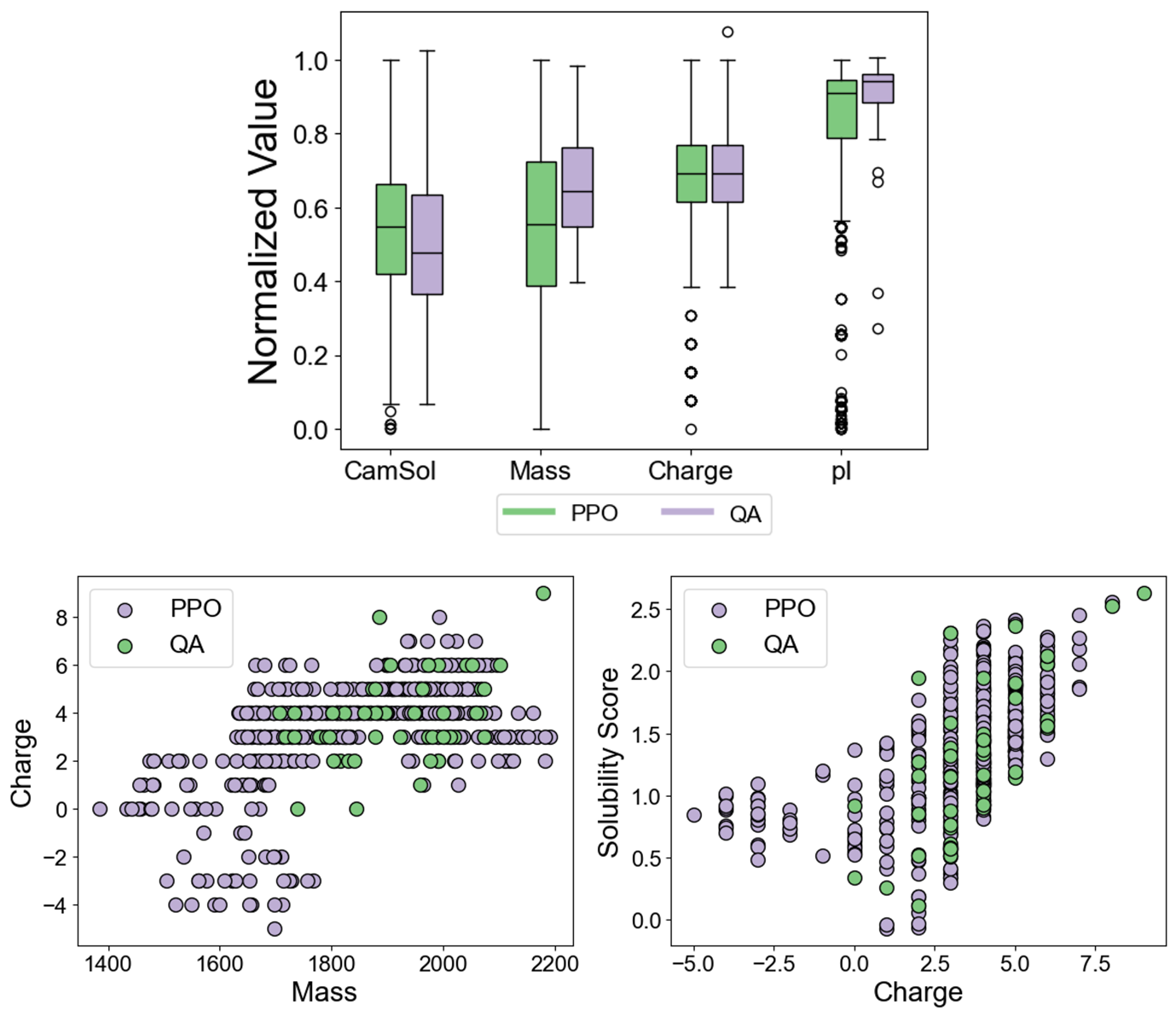


**Figure S2. Comparison of physicochemical properties of peptides discovered using quantum annealing (QA) or proximal policy optimization (PPO).**

Figure S2 compares physicochemical properties of QA or PPO peptides for polyethylene. The properties investigated are the CamSol solubility score, the peptide mass, the peptide charge at pH 7, and the isoelectric point (pI). The top panel compares the range of properties for QA and PPO designs, with values shifted and normalized such that the minimum and maximum values for the QA peptides ranges from 0 to 1 (raw values are provided in the data file). Notably, PPO peptides sample a larger range of mass, charge, and pI values than PPO peptides. The bottom two panels show a 2D plot of [mass, charge] pairs (left) and [charge, solubility] paris (right) for PPO and QA peptides, with each point representing one peptide. In accordance with the top figure, PPO designs sample more pairs of properties. These results indicate that PPO was able to find PBPs with more diverse physicochemical properties.


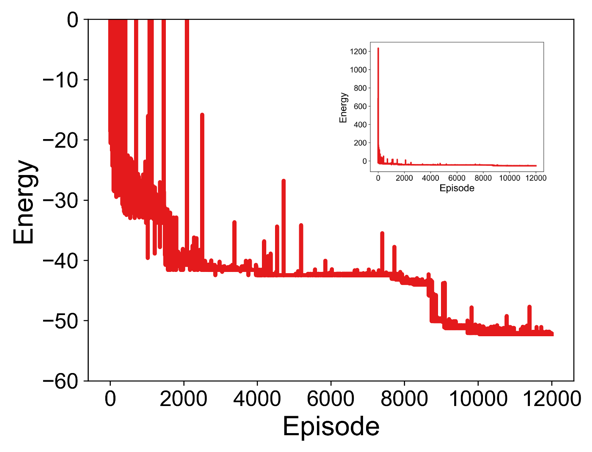


**Figure S3. Example trajectory of peptide score (energy) over course of PPO.**

Figure S3 underscores the significance of initializing the policy network in the generation of new peptide sequences. By utilizing a pre-trained policy network, a diverse set of peptides is created through iterative modifications to approach an optimal sequence. The figure illustrates the variance in the number of iterations required to attain a benchmark PepBD score when initiating the peptide generation process from a known optimal amino acid sequence, as determined by quantum annealing (QA), versus beginning with a random sequence. It is observed that starting with the QA-derived optimal sequence leads to convergence at the optimal point with fewer iterations.


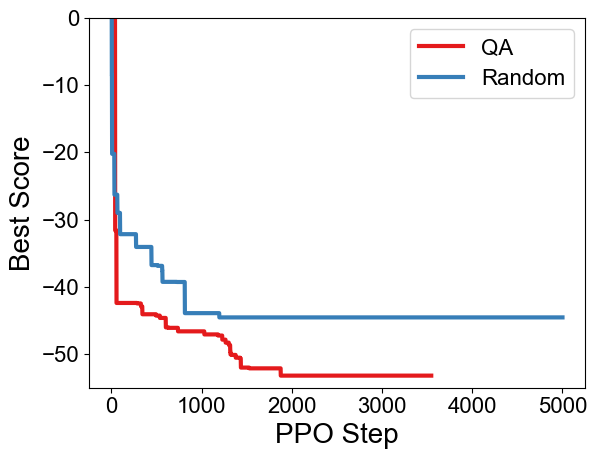


**Figure S4. Using Best QA design as starting point for PPO improves PPO output.** Result is shown for system conformation 3 for polyethylene.

Figure S4 evaluates the impact of the starting sequencing for deploying PPO. A PPO policy is learned by attempting to learn how the score will change upon modifying a given peptide sequence. Figure S4 compares PPO output for two different starting sequences: a random amino acid sequence or the best QA sequence for the same peptide structure. The plot shows the best score at each PPO step for both starting sequences. A much lower score is found when using QA as the starting point than when using a random sequence as the starting point.


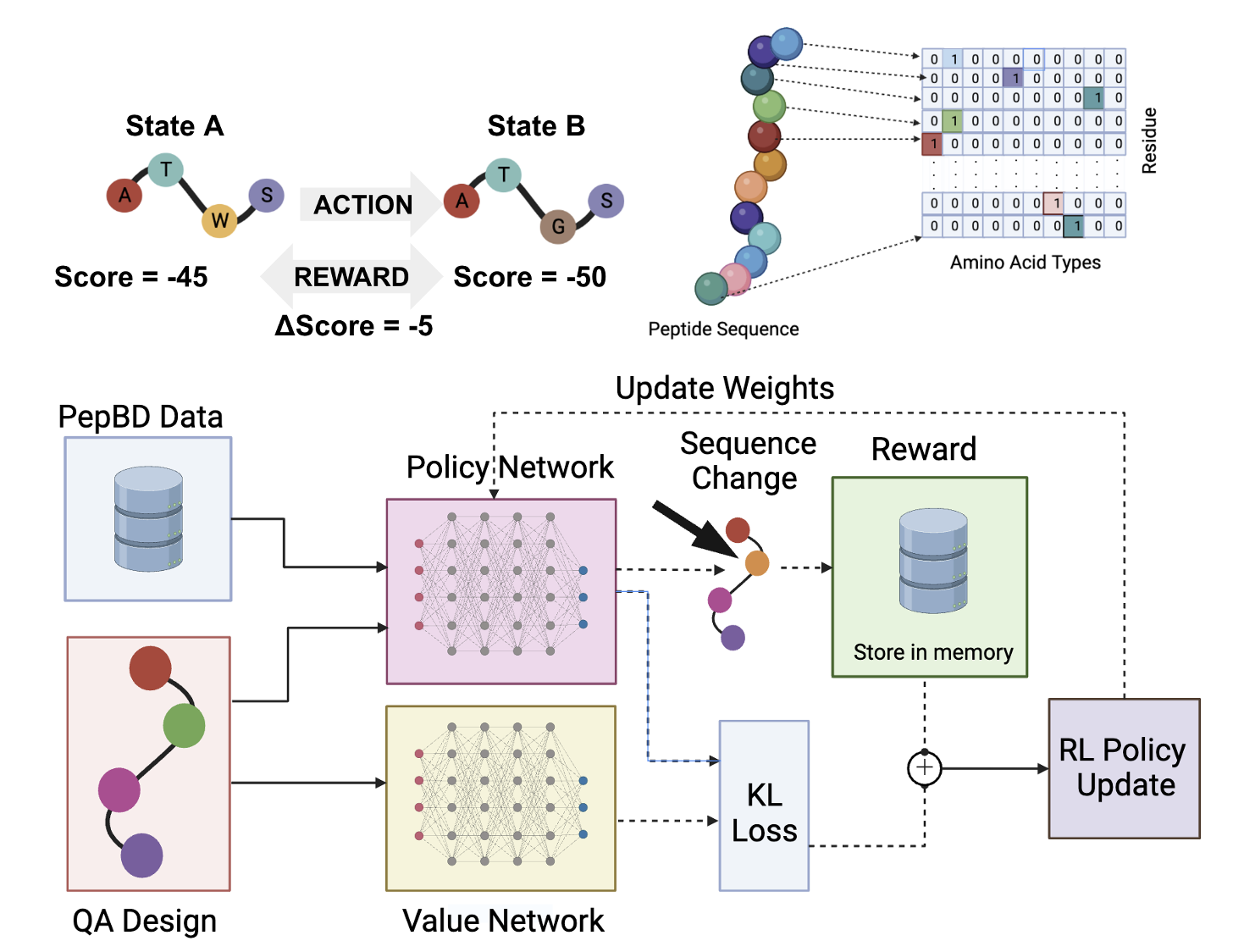


**Figure S5. Top left:** For peptide optimization using RL, the state is the peptide sequence, actions are changes to the peptide sequence, and rewards are the change in the predicted PepBD score. **Top Right:** Peptides are represented using a one-hot encoding based on amino acid sequence. **Bottom:** RL model used for peptide optimization. The input is the best sequence found by quantum annealing (QA), the policy network is trained on PepBD data, the loss is the Kullback-Liebler (KL) divergence between the policy and value network, and the RL policy is updated based on the KL loss and the reward upon changing the peptide sequence. An example trajectory of the QUBO score during PPO is shown in Fig. S3. Figure made using Biorender.

Figure S5 illustrates the architecture utilized for Proximal Policy Optimization (PPO). All 19 amino acids are encoded using one-hot encoding. Rewards are calculated based on the PepBD score, which integrates both single-body and two-body terms to evaluate the total affinity of interaction with the surface and environment. In each iteration, the action dictated by the learned policy results in the modification of a single amino acid at a specific residue. Feedback is subsequently recorded as a reward, which is determined by the difference in the PepBD scores between the modified sequence and the original sequence. A Gated Recurrent Unit (GRU)-based network is employed for both the policy and value networks. The standard Proximal Policy Optimization algorithm is applied to navigate the potential solution space within a fixed conformation.

**Table S1. Statistical comparison of amino acid frequencies between QA designs for different plastics.** The plastics are polyethylene (PE), polypropylene (PP), polystyrene (PS), and polyethylene terephthalate (PET). P-values were calculated by performing a two-tailed t-test with unequal variance. The dataset was the number of times each amino acid type appeared in a peptide for all quantum annealing (QA) designs. Entries are red if the difference is statistically significant at the 0.05 threshold and the amino acid frequency is less than 5% for both plastics (i.e. result is statistically significant, but no practical effect). Entries are blue are blue if the difference is statistically significant at the 0.05 threshold and the amino acid frequency is greater than 5% for at least one of the plastics (i.e. result is statistically significant and may have a practical effect). Entries are listed as “NA” if the amino acid never appeared in a design for both plastics.

| **Amino Acid** | **PE vs PP** | **PE vs PS** | **PE vs PET** | **PP vs PS** | **PP vs PET** | **PS vs PET** |
| --- | --- | --- | --- | --- | --- | --- |
| R | **2.12E-04** | **2.84E-03** | **4.70E-04** | 4.58E-01 | 8.28E-01 | 6.47E-01 |
| H | 8.23E-01 | 1.04E-01 | 7.68E-02 | 3.02E-01 | 2.54E-01 | 8.88E-01 |
| K | 7.03E-01 | **6.11E-03** | 1.01E-01 | 1.33E-01 | 4.25E-01 | 5.75E-01 |
| D | **3.11E-02** | 1.21E-01 | 3.11E-02 | **0.00E+00** | NA | 4.29E-01 |
| E | 1.69E-01 | 8.18E-01 | 5.65E-01 | **0.00E+00** | 0.00E+00 | 4.62E-01 |
| S | 1.63E-01 | 4.36E-01 | 1.63E-01 | **0.00E+00** | NA | 4.29E-01 |
| T | 6.29E-01 | 1.49E-01 | 6.29E-01 | 5.50E-01 | 1.00E+00 | 6.30E-01 |
| N | 5.13E-01 | 7.63E-01 | 6.51E-01 | 7.02E-01 | 8.27E-01 | 8.54E-01 |
| Q | 6.11E-01 | 6.11E-01 | 8.99E-01 | 1.00E+00 | 7.39E-01 | 7.52E-01 |
| C | 1.69E-01 | 6.00E-02 | 4.90E-01 | 5.44E-01 | 4.19E-01 | **0.00E+00** |
| G | **1.45E-03** | 7.54E-02 | **3.11E-02** | 1.24E-01 | 2.54E-01 | 7.55E-01 |
| P | NA | NA | NA | NA | NA | NA |
| A | 1.44E-01 | 1.44E-01 | 1.44E-01 | NA | NA | NA |
| I | 7.94E-01 | 2.41E-01 | 5.14E-01 | 2.25E-01 | 4.38E-01 | 7.69E-01 |
| L | 4.98E-01 | 7.34E-01 | **3.62E-02** | 3.04E-01 | 1.48E-01 | 1.08E-01 |
| M | 9.12E-02 | 7.34E-01 | 3.08E-01 | 9.78E-02 | 4.03E-01 | 5.62E-01 |
| F | 1.33E-01 | 1.10E-01 | 1.92E-01 | 1.21E-01 | 1.61E-01 | 7.66E-01 |
| W | 4.43E-01 | 2.80E-01 | 6.52E-01 | 1.70E-01 | 8.13E-01 | 1.67E-01 |
| Y | 4.01E-01 | 5.29E-01 | **5.45E-04** | 8.39E-01 | **9.41E-05** | **2.47E-04** |
| V | 5.70E-01 | 1.00E+00 | 5.70E-01 | 6.57E-01 | 1.00E+00 | 6.92E-01 |

Table S1 provides a statistical analysis of the differences in the amino acid compositions of peptides designed by quantum annealing (QA) for the plastics polyethylene (PE), polypropylene (PP), polystyrene (PS), and polyethylene terephthalate (PET). The majority of the entries are black, indicating the amino acid frequencies do not differ significantly between plastics in the majority of cases. When statistically significant differences were found, many of them were for amino acids that comprised less than 5% of the amino acids (red entries) for both plastics, indicating the difference is statistically significant but not practically significant. The remaining blue entries show significant differences between plastics for amino acids that comprise more than 5% of the amino acids for at least one of the plastics. These differences are thus likely both statistically and practically significant.

Dataset S1 (separate file). Contains Potts model energies, QA and PPO peptides, properties of discovered peptides, and MD simulation data.
